# A Byzantine Sensing Network Based on Majority-Consensus Data Aggregation Mechanism

**DOI:** 10.3390/s21010248

**Published:** 2021-01-02

**Authors:** Jenghorng Chang, Fanpyn Liu

**Affiliations:** 1 School of Defense Science, CCIT, National Defense University, Taoyuan 335009, Taiwan; 2 Computer Science and Information Engineering, CCIT, National Defense University, Taoyuan 335009, Taiwan; phoebeliu@ndu.edu.tw

**Keywords:** wireless sensor network, byzantine agreement, majority function, consensus

## Abstract

In the current Internet of Things era, digital devices form complex interconnections. The statuses of objects of interest are monitored using sensors, and distributed wireless sensor networks are formed from numerous sensor nodes. Many Byzantine fault tolerance mechanisms in wireless sensor networks (WSNs) were proposed from Byzantine agreement which even with a few faulty nodes in a sensor network, most healthy nodes can reach a consensus, perform data transmission tasks, and maintain network operation. In this study, this mechanism was utilized together with the majority function technique; in particular, the proposed method uses original sensor signals to define a threshold to assert a binary value of one or zero, thereby performing data judgment and aggregation. This approach reduces node energy consumption and enables the nodes to quickly reach a consensus. Moreover, the operating performance of the network can be maintained even when problems such as node failure and faults occur within the fault tolerance range. Compared with existing algorithms, the proposed data aggregation mechanism exhibits a better network life cycle and can effectively extend the flexibility of network operations.

## 1. Introduction

A wireless sensor network (WSN) [1] is a large-scale distributed system used for sensing and processing space-intensive data. Most WSNs employ large numbers of nodes to perform security/monitoring tasks, environmental monitoring, health monitoring, industrial automation, disaster management, and other challenging tasks in harsh environments. Although node resources are limited, complex tasks, such as decentralized detection and evaluation [2], can be achieved through node cooperation.

The Internet of Things (IoT) is a relatively new concept in the field of information technology [3]. Many information devices can form end-to-end links with a server through suitable communication protocols (e.g., Wi-Fi, ultra-wideband, Bluetooth, and ZigBee), thereby expanding applications in the fields of industrial monitoring, smart technology, and home care, among others. By combining the IoT with WSNs, the scope of management can also be extended effectively.

Whether it is WSN or IoT, which a distributed system has three significant consequences, such as concurrency, no global clock, and independent failures [4]. Because of above characters, a lot of research proposed many methods to allow some nodes fail and the others still operating. Since many applications in low-power wireless networks require complex coordination between their members (nodes). Such applications require consensus algorithms to enable coordination within short periods of time to execute tasks. While consensus has been studied for wired networks decades ago, with, for example, Paxos and Raft, it remains an open problem in multi-hop low-power wireless networks due to the limited resources available and the high cost of established solutions [5].

However, once a large number of information devices (referred to as sensor nodes) in the WSN encounter failures or faults, the network may not operate normally. In severe cases, the entire network may be shut down. Therefore, in early WSN environments, large numbers of nodes were deployed to reduce the effects of node failures on network operations. Considering such an environment, a fault-tolerant data aggregation mechanism was designed in this study to tolerate the faults of a few sensor nodes in a network, such that network operation can be maintained and valid data can still be acquired. Moreover, by quickly arriving at a consensus and transmitting data among the nodes, the network stability can be improved effectively and the network life cycle can be extended.

Thus, this paper proposes a method for optimizing the network life cycle and the number of surviving nodes based on the Byzantine fault tolerance mechanism. By using the principle and the characteristics of the majority-consensus algorithm, we also simulate a clustered network that provides both a fault tolerance and a data aggregation mechanism.

The remainder of this paper is structured as follows. A literature review is provided in Section 2, the proposed method is explained in Section 3, the experimental analysis is detailed in Section 4, and the conclusions and future research directions are presented in Section 5.

## 2. Literature Review

### 2.1. WSN

WSNs originated from a project for military applications, conducted by the University of California, Berkeley, where researchers used micro-electromechanical systems technology to develop a button-sized sensor called “Smart Dust” [6]. This type of sensor is used in battlefields to monitor and provide warnings regarding the whereabouts of enemies. The data collected from the sensors are sent back to a sink, through a wireless network, for aggregation and analysis. The obtained intelligence then serves as a reference for subsequent operations.

Arampatzis et al. [7] pointed out that WSNs based on IoT have been widely used in the military, homeland security, medical care, ecological conservation, agricultural monitoring, and manufacturing. By deploying a large number of sensors in a specific range, the relevant data regarding objects of interest can be collected and subsequently analyzed and identified. Because the IoT must respond and take action in response to changes in the surrounding environment, it is critical to have a fault-tolerant and reliable data transmission mechanism.

In a paper on WSNs, Handy et al. [8] proposed a low-energy adaptive clustering hierarchy (LEACH) algorithm. The algorithm generates clusters in each working round and randomly selects cluster heads (CHs) from nodes, which are responsible for transferring sensor data in each cluster to the sink. Li et al. [9] improved the LEACH algorithm and proposed a distributed energy-efficient clustering (DEEC) algorithm. The DEEC algorithm considers the remaining energy of nodes when selecting CHs, preventing the quick deaths of low-energy nodes because they serve as CHs for a long time. Since then, many scholars [10,11,12,13,14,15] have proposed various methods entailing WSNs, such as SEP, PEGASIS, and HEED, to save energy and handle greater workloads; these methods extend the lives of sensor nodes and achieve the best operating efficiency in an environment with limited energy. In addition, some researchers [16,17,18] have surveyed and compared the aforementioned methods. The most well-known WSN algorithms are shown in Table 1.

### 2.2. Byzantine Agreement (BA)

BA is a well-known fault-tolerant method. Lamport et al. [19] quoted the historical example of consensus among the generals of the Byzantine Empire with regard to attacking their enemies and applied this concept to develop BA. Specifically, for a reliable computer system to troubleshoot processor faults effectively, the following conditions of the consensus protocol must be met:

- Agreement: All processors agree on a common value.
- Validity: All processors agree on the initial value sent.
- Termination: All processors reach a final decision.

To rule out cases in which the system operation failure is caused by only a few processor faults (e.g., failures and hacker intrusions), Lamport et al. [19] proposed a fault tolerance threshold:

*f* ≤ ⌊*n* − 1/3⌋ (1)

where *f* is the number of faults and *n* is the total number of processors.

Since then, with the rapid development of computer and network systems, many scholars [20,21] have proposed various improved fault tolerance thresholds based on the BA from different perspectives with the objectives of reducing the incidence of failures and improving the system operating efficiency, as shown in Table 2.

Moniz et al. [20] proposed the Turquois algorithm for wireless ad hoc networks, which can tolerate *f* faulty processes while allowing *n* processes on the network, that is, *f ≤ n/*3. The network is allowed to operate normally when *σ ≤* ⌈*n* *− t/*2⌉*(n* *− k* *− t) + k* *−* 2, where *σ* denotes the number of omission faults, and a consensus is finally reached. Rabin et al. [21] referred to BA, proposed by Lamport et al. [19], and proposed a solution that can reach a final consensus in synchronous and asynchronous systems containing *t(t ≤ n/*4*)* faulty processes, where the security of message transmissions is verified using digital signatures. In this paper, we wanted to combine the BA and majority function to observe the phenomenon in the distributed sensor network.

### 2.3. Consensus

Consensus is a concept equivalent to group decision. In this concept, the group participants can exchange message and decide some policies. Similarly, when we apply this concept to WSNs, each node can negotiate and cooperate with each other to achieve the transmission of network data.

A consensus can lead to decisions, and the majority-consensus (MC) algorithm is a majority decision-making algorithm. It was used as a solution to update distributed databases in the 1980s. Even if some databases (minority) are disconnected, the database replicas (majority) can be updated with regard to the remaining databases, keeping the data consistent and updated [22].

Currently, the MC algorithm constitutes part of the data processing method employed in sensor network application development modules. Through a threshold value, the collected sensing data are asserted (asserted/deasserted) with binary values of one or zero at the sensor nodes; then, in accordance with the MC concept, the final consensus is calculated and sent to the sensor network sink [23].

Gogolev et al. [24] proposed a distributed binary majority-consensus rule to study different reactions to disturbances. When a network has different kinds of disturbances, such as message loss and stochastic noise, the random neighbors majority (RM) also involves better tolerance toward the influence of faulty nodes.

Fischer et al. [25] proposed a consensus method that allows systems to make errors and reach a final decision, where the binary values of one and zero are used for error judgment. In this study, the method described in the literature [26,27] was referenced. Accordingly, threshold assertion was performed based on a binary value and incorporated into the fault tolerance mechanism, a WSN environment was simulated, and data were aggregated from sensor nodes to reach a final consensus.

### 2.4. Majority Function (MF)

Logic gates play an important role in digital logic circuits. These gates combine the high and low voltages of transistors (i.e., “1” and “0” bit values) to represent logical TRUE and FALSE states [26]. A Majority Gate (MG) is based on the input state. That is, if more than half of the input values are “1,” then the output value *F* is “1”; otherwise, the output value is “0.” In addition, when a gate has several inputs and one output, it may form a MF [27]. The corresponding formula is as follows:

(2) $$Majority\left(p_{1},\ldots ,p_{n}\right)=\lfloor \frac{1}{2}+\frac{\left(\sum_{i=1}^{n}p_{i}\right)-1/2}{n}\rfloor$$

This paper proposed a novel theory in which the BA fault tolerance mechanism is considered and the aggregated sensor data are passed through a threshold to assert a binary value of “1” or “0.” Khan et al. [28] proposed a cooperative theory that every clusters deploy a coordinator as the Cluster Head (CH), which have strong computing and full functions, communicating with other device and calculating the consensus degree. In this paper, the MC calculation is performed on each cluster, and the MC result is sent by the respective CH to the sink, which completes the aggregation of the sensor data. The MC calculation is then performed again to ascertain the validity of the data within the sensing range.

## 3. Research Method

The method proposed in this paper, i.e., the Byzantine consensus algorithm (BCA), was developed with reference to the research of Lamport et al. [19]. It is based on the Byzantine fault tolerance mechanism and uses MC to aggregate sensor data. Moreover, we refer to Wang et al. [29] to propose some assumptions clearly. The proposed method is based on the following basic assumptions:

- In a distributed network, the total number of nodes is a constant *n*(*n ≥* 4), which is minimum requirement.
- Each node communicates with the other nodes in a reliable and fully connected network environment and performs sensing tasks with limited energy *e*.
- One or more nodes may encounter faults or failures; a faulty node will send an error message to the other nodes, but a failed node will not send any messages.
- After the message exchange, when the number of faulty or failed nodes *f* is less than the fault tolerance value corresponding to the total node number *n* (e.g., *f* < *n*/3), the network will maintain normal operation.
- When the number of faulty or failed nodes is less than the fault tolerance value, the MC of the sensing data of the nodes *d* will be calculated.

Some target areas desolate, dangerous or inaccessible to humans there are many challenges for surveillance and monitoring by deploying and maintaining a large number of static nodes. This paper presents a fault-tolerant data aggregation mechanism for clustering sensor network to solve this problem. In this paper, the clustering sensor network can extend its operating time and maintain operational flexibility, and we use the energy-saving mechanism of the cluster to decentralize the management of sensor nodes and allow a small number of nodes to fail. In addition, our research is different from dynamic networks, in which nodes must consider factors such as moving time and offset distance. To make this research consistent with an actual wireless sensing environment, initial energy values are assigned to the sensor nodes based on the characteristics of actual nodes with limited energy, and the energy of the nodes is increasingly consumed as the number of experimental rounds increases.

**Figure 1:**
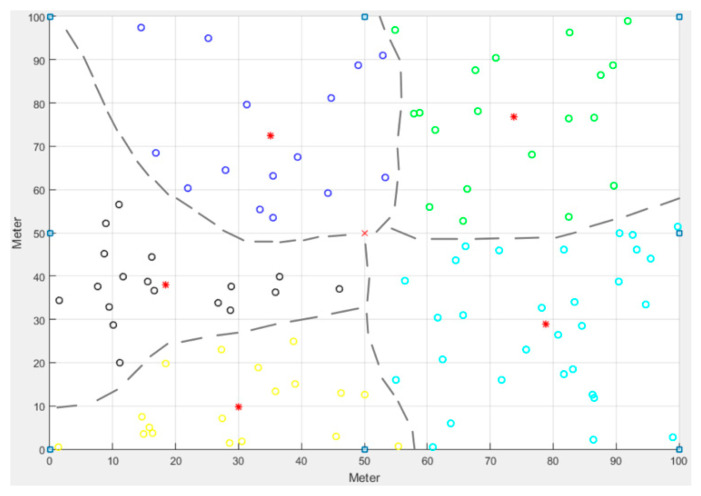
Schematic diagram of K-means network topology (cluster number = 5).

K-means [30] is a clustering algorithm employed in machine learning. Several scholars [31,32] in the field of WSN research have attempted to obtain the optimal clustering number by improving this algorithm, so as to reduce the energy consumption of sensor nodes and extend the network life cycle. The K-means clustering algorithm was also adopted in the present study to establish a clustered network architecture, as shown in Figure 1, aggregate the MC results from the clusters and send them to the sink through the CHs, and complete the data aggregation from the CHs.

In Section 4, three methods—LEACH, DEEC, and SEP—are compared with the proposed method. The experimental comparison results are then used to analyze the network life cycle and node energy consumption performance.

### 3.1. Initial Network Setup

The initial energy values of nodes were set in the initial stage of this experiment. Further, “Node” represents the total number of nodes *n*, *N_i_* represents the *i*th node, and *N_i_^energy^* is the energy *e* of the *i*th node. It was assumed that the energy of the nodes decreased as the number of experimental rounds increased.

### 3.2. MC Calculation at the CHs

In Algorithm 1, *N_i_^inital^* represents the values sensed by the sensors, which are generated randomly based on environmental monitoring indicators. Furthermore, the assertion value is determined for the threshold *T* and is calculated as zero or one, as specified in the assertion policy. Indicator values below the threshold value are set as zero and vice versa. *N_i_^binary^* represents the binary value after node *N_i_^inital^* is asserted. The binary value corresponding to each node is then aggregated by the corresponding CH to produce the cluster majority result, denoted as *Majority _Result* (Equation (3)). Algorithm 1 is utilized to calculate the MC of the nodes in each cluster, producing the results of this stage.
**Algorithm 1** BCA (cluster MC calculation stage)Input: Initial value of nodes *N_i_^inital^*
Output: Majority result of each cluster (*Majority_Result*)1 random(*N_i_^inital^*)  //Randomly generate the values sensed by nodes according to environmental monitoring indicator values. 2 if 0 < *N_i_^inital^* < *T*, then *N_i_^binary^* = 0  //When the value sensed by a node is less than the threshold T, the corresponding assertion value is 0. 3 else *N_i_^binary^* = 1  //When the value sensed by the node is greater than the threshold, the assertion value is 1. 4 if ∑*N_i_^binary^* ≥ *n*/2, then *Majority_Result* = True  //When the final majority result is greater than or equal to half the total number of nodes, an MC is reached. 5 else ∑*N_i_^binary^* < *n*/2, then *Majority_Result* = False //When the final majority result is less than half of the total number of nodes, no MC is reached

### 3.3. Consensus Calculation for the Entire Network

After the *Majority_Result* values corresponding to all clusters have been obtained through the MC calculations at the CHs, the distances between clusters must be considered. If majority results are exhibited by neighboring clusters in a particular region, then there is a high consensus among the sensor data in that region. Conversely, if majority results occur in scattered clusters in a region, then the consensus level among the sensor data is relatively low in that region.

Therefore, when the distribution of clusters in the entire network is not considered, factors such as the distance between the clusters and degree of clustering may affect the final consensus results of the network, resulting in failure to provide reliable sensor data. In this study, the SOP operation is performed to further calculate the consensus results for the entire network and verify the data reliability. Accordingly, Algorithm 2 is proposed.
**Algorithm 2** BCA (network consensus calculation stage)Input: Majority result of each cluster (*Majority_Result*) Output: Sensor network consensus result (*Consensus_Result*)1 if ∑ *Majority_Result* = False, then break,  //When the final consensus result is false, return to a new round. 2 else if ∑ *Majority_Result* = True & SOP = True, then *Consensus_Result* = True,  //When the final consensus result is true, a consensus is reached. 3 else if ∑ *Majority_Result* = True & SOP = False, then *Consensus_Result* = False, //When the final consensus result is false, no consensus is reached.

In Algorithm 2, the SOP operation uses the majority result obtained from each CH as the input, Equations (2) are employed to perform the SOP operation, and the obtained result is either one or zero. Then, the MC result of the entire network is calculated at the sink, and the final output is *Consensus_Result*.

For compliance with the BA fault tolerance mechanism, testing for faults must be performed in each round of sensing to ensure the validity of the final consensus. When nodes in the network environment encounter problems, such as death or non-responsiveness, they will be regarded as faulty nodes. Once the total number of faulty nodes is greater than or equal to the fault tolerance value, the sensor network will stop operating.

In summary, when all nodes have collected sensing data, assertions will be made. Then, the MC will be calculated. Finally, the sensing results that can exhibit Byzantine consensus will be obtained. The complete process is as follows:

1. Divide clusters using the K-means clustering algorithm.
2. Confirm whether the number of network faulty nodes is greater than or equal to the fault tolerance value.
3. Collect sensing data at the sensor nodes in each cluster.
4. Assert the collected sensing data through a threshold to obtain a binary value of “1” or “0.”
5. Calculate the MC of nodes in each cluster at the respective CH.
6. Calculate whether the binary value of each CH reaches an MC via the SOP operation at the sink.
7. The entire network reaches an MC, and each cluster sends the sensing data to the sink.

## 4. Experimental Simulation and Analysis

In this study, we used MATLAB 2015 (MathWorks, Natica, Massachusetts, USA) [33] to implement the simulation environment. First, we assumed an experimental sensing environment with a size of 200 m × 200 m to analyze the network life cycle and other experimental values.

Moreover, by referring to the first-order radio model [34], the radio energy consumption was used as the energy consumption index for radio transmission and reception. The cluster center generated by the K-means clustering algorithm was regarded as the CH, which aggregated the sensing data of each node in the cluster to determine the cluster consensus value and sent the result to the sink.

The initial parameter settings of the experiment are listed in Table 3. First, we deployed 100 fixed sensor nodes and a sink within our simulation network. In this study, three, five, and seven clusters were used for the experimental analysis. The number of clusters was determined according to the majority function (MAJ) [35]. Equation (3) shows that the number of clusters *n* takes three or more odd numbers as the input, and *C_i_* is the numbering of the clusters.

(3) $$\mathrm{MAJ}\left(C_{1},C_{2},\ldots ,C_{n}\right)=\{\begin{array}{ll} 1, & if\sum_{i=1}^{n}C_{i}\geq \frac{n}{2}\\ 0, & otherwise \end{array}$$

Furthermore, we added different fault tolerance values to observe the entire life cycle and number of surviving nodes changing in our experiments.

### 4.1. Experimental Environment Simulation

The simulated experimental environment of this study constituted a 200 m × 200 m sensor field; the sensor nodes were divided into three, five, and seven clusters and the majority result of each cluster is calculated; further, 2000 rounds of experiments were executed and obtained final consensus results from each round. Moreover, based on the experimental results, the entire life cycle and number of surviving nodes were analyzed and compared.

### 4.2. Experimental Data Analysis

#### 4.2.1. Nonconsideration of Fault Tolerance Conditions

**Figure 2:**
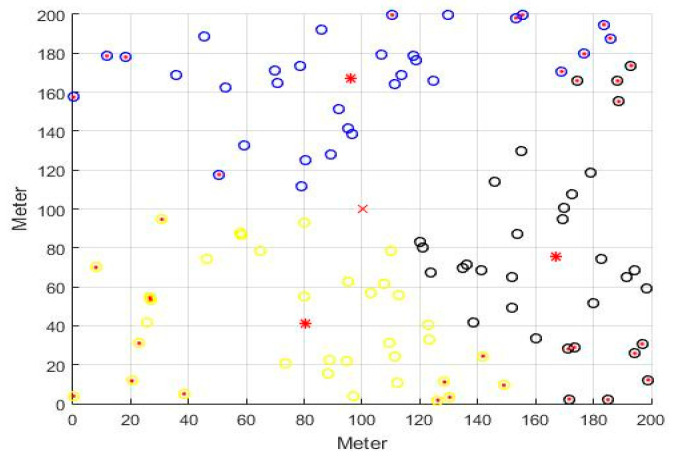
Schematic diagram of nodes throughout the network.

In accordance with the parameters given in Table 3, experimental simulations with three, five, and seven clusters were conducted to obtain the entire network life cycle and the number of surviving nodes. Figure 2 shows the status of dead nodes in the entire network after 2000 rounds of experiments with three clusters. The red multiplication symbol (×) represents the sink position, the red asterisk (*) represents the CH, the solid red dots are the dead nodes, and the remaining hollow dots are the surviving nodes.

**Figure 3:**
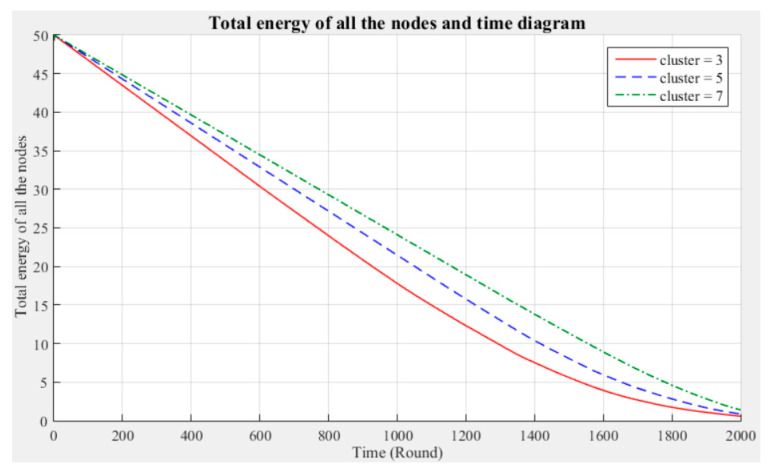
Entire network life cycle (200 m × 200 m).

**Figure 4:**
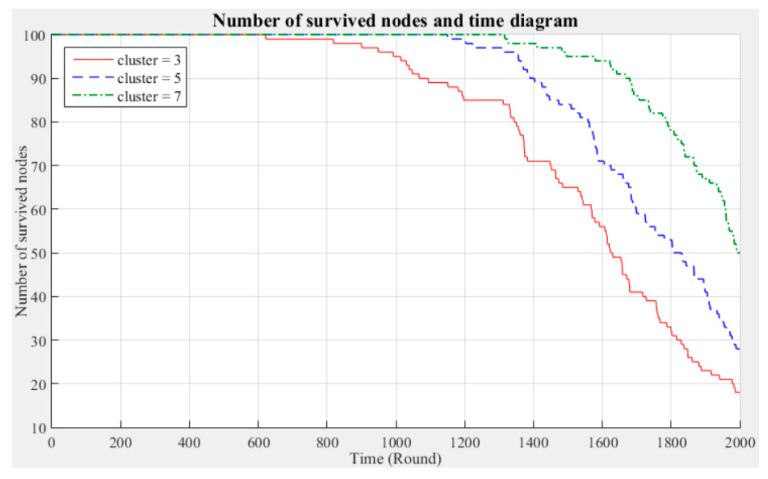
Number of surviving nodes in the network (200 m × 200 m).

Figure 3 and Figure 4 respectively compare the entire network lifecycle and the number of surviving nodes in the cases with three, five, and seven clusters, without considering the Byzantine fault tolerance. As shown in the figures, when the Byzantine fault tolerance is not considered, the entire network has a longer life cycle and more surviving nodes within 2000 rounds when seven clusters are employed. The network life cycle and number of surviving nodes obtained with three clusters are the most unfavorable. The experimental results show that in a network environment with a small number of clusters, the energy consumption will also be greater because of the large data transmission distances between the nodes and CH.

#### 4.2.2. Consideration of Fault Tolerance Conditions

Similar to the previous experiment, three, five, and seven clusters were used again, and Byzantine fault tolerance values of one-half, one-third, and one-quarter were considered to reach a Byzantine consensus. The effects of different fault tolerance values on the entire network life cycle and the number of surviving nodes were then analyzed.

**Figure 5:**
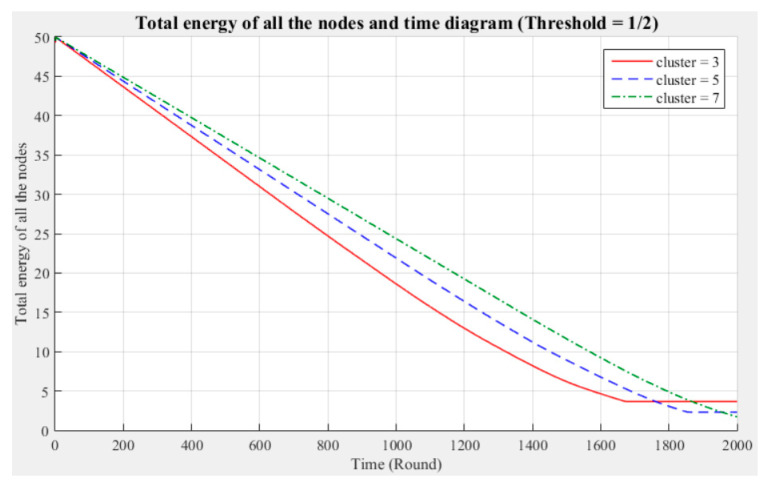
Entire network life cycle (fault tolerance = 1/2).

**Figure 6:**
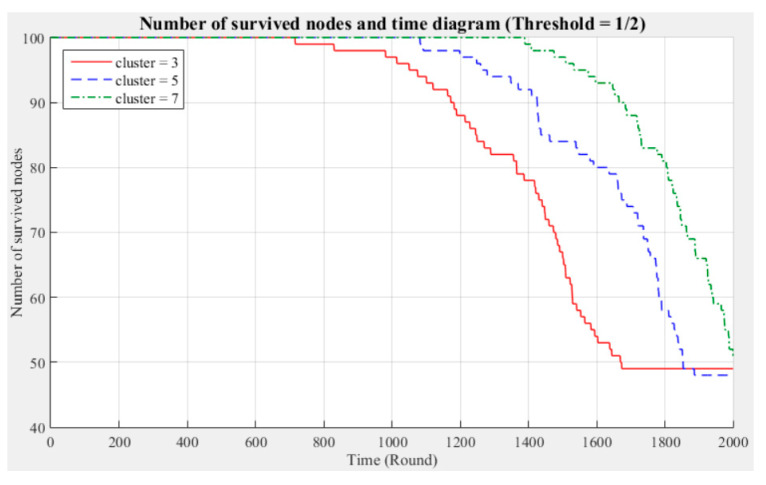
Number of surviving nodes in the network (fault tolerance = 1/2).

**Figure 7:**
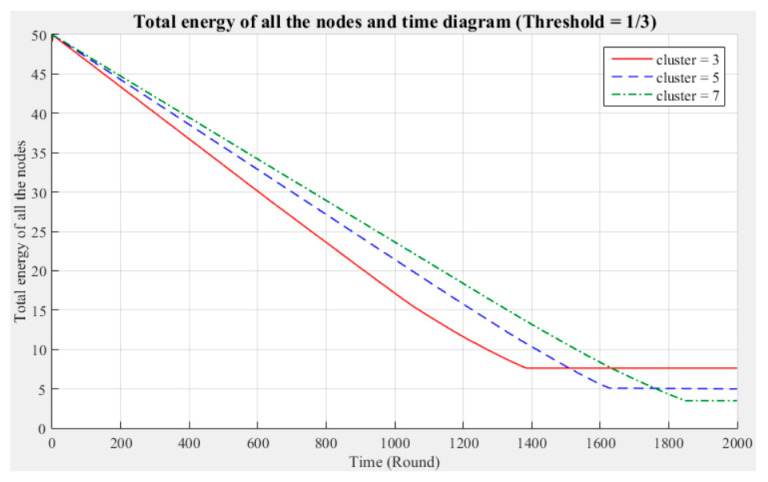
Entire network life cycle (fault tolerance = 1/3).

**Figure 8:**
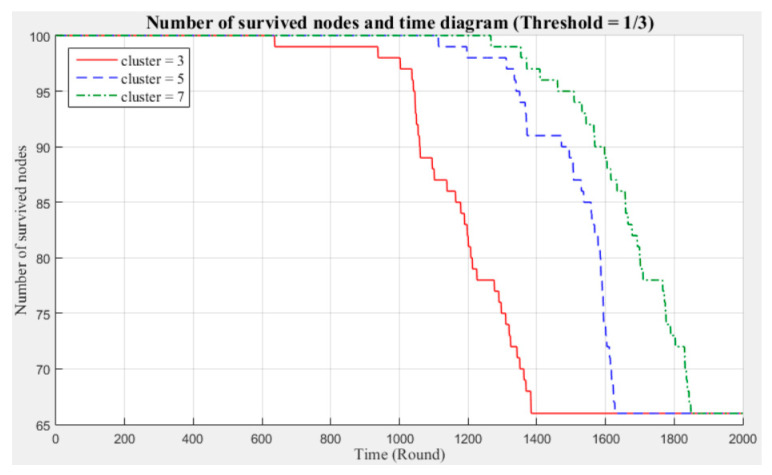
Number of surviving nodes in the network (fault tolerance = 1/3).

**Figure 9:**
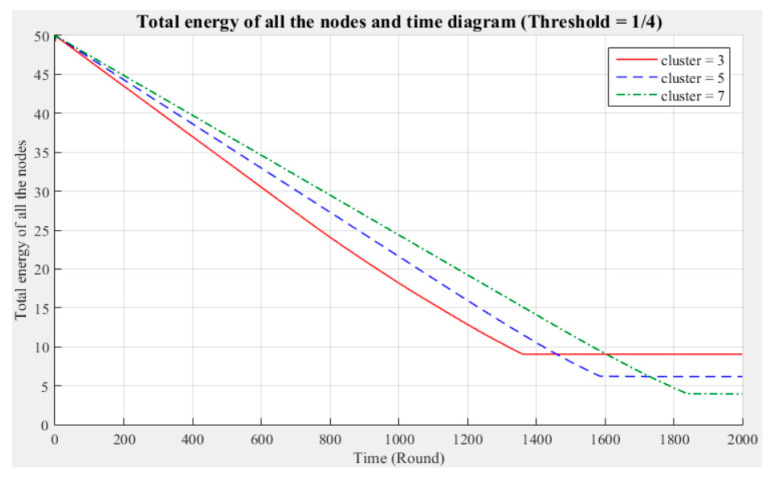
Entire network life cycle (fault tolerance = 1/4).

**Figure 10:**
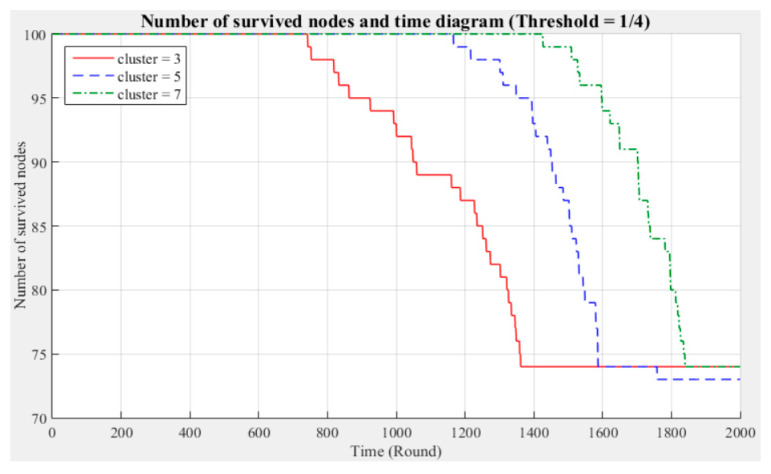
Number of surviving nodes in the network (fault tolerance = 1/4).

Figure 5, Figure 6, Figure 7, Figure 8, Figure 9 and Figure 10 show the network (100 sensor nodes) life cycles and number of surviving nodes determined from 2000 rounds of experiments under different cluster numbers and fault tolerance values.

The following phenomena are observable in the experimental results:

- Network life cycle: The final remaining energy of the network shows that three clusters cause the nodes to consume energy faster and the network to stop working in the early rounds.
- Changes in the surviving nodes: The number of faulty nodes reaches the fault tolerance value fastest when there are three clusters, which indicates that the nodes in each cluster consume more energy, resulting in a lower node survival rate.
- Different fault tolerance values: Under different numbers of clusters, when the fault tolerance value is one-half, the node survival rate is optimal. This finding indicates that the sensor network can maintain the optimal network operation efficiency by adopting a less rigorous fault tolerance condition.

For the above experiment, Figure 5, Figure 6, Figure 7, Figure 8, Figure 9 and Figure 10 show that in a network with more clusters, the nodes are closer to the CH and the energy consumption is less, which can enable the maximum workload for the entire network. However, in a network with fewer clusters, the nodes are farther from the CH and the energy consumption is greater; hence, the entire network has a relatively poor operating efficiency. These results show that the number of clusters is a key factor affecting the network operation and that better performance is associated with a larger number of clusters.

#### 4.2.3. Sink Deployment Environment

**Figure 11:**
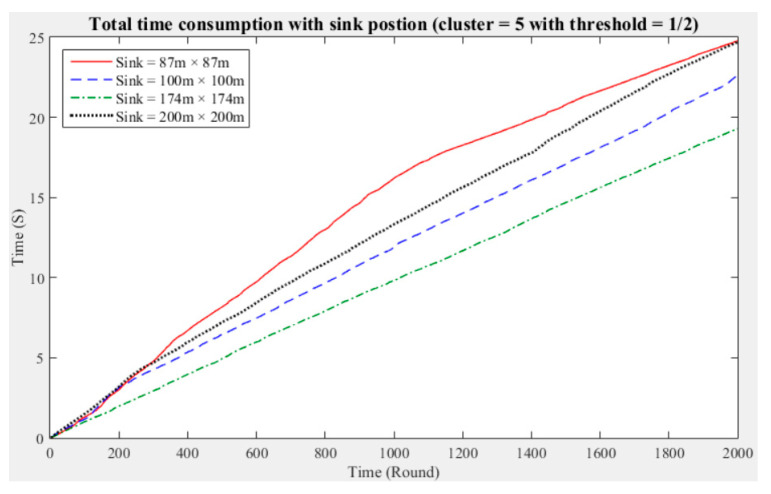
Data aggregation time consumption for the entire network (cluster number = 5, fault tolerance = 1/2).

**Figure 12:**
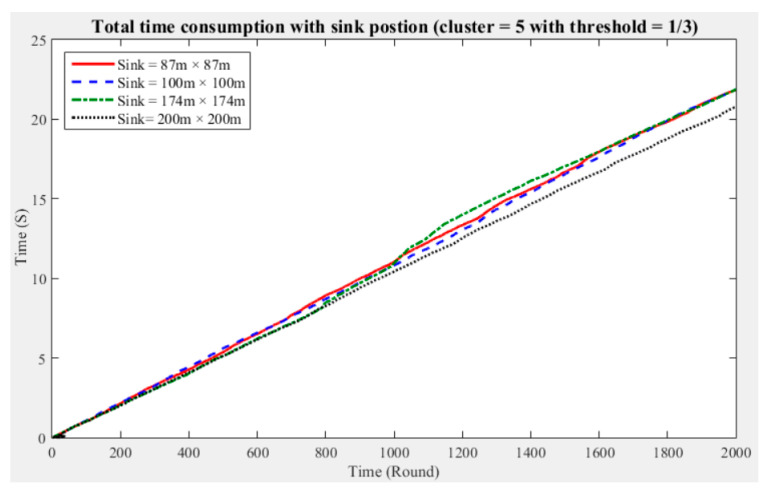
Data aggregation time consumption for the entire network (cluster number = 5, fault tolerance = 1/3).

**Figure 13:**
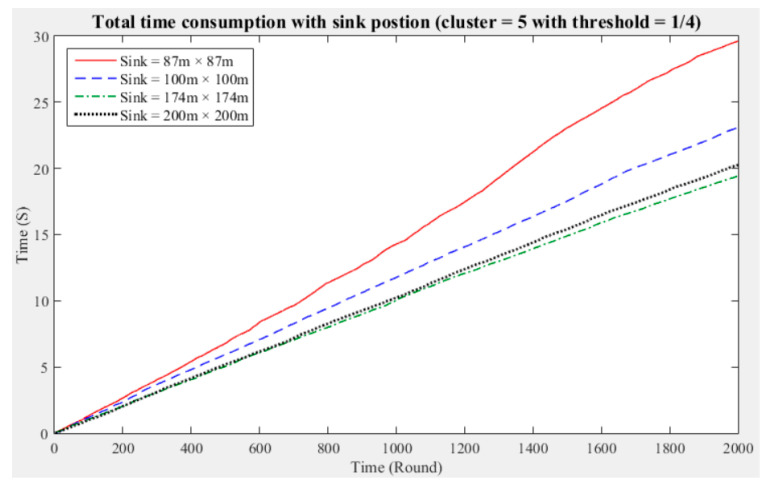
Data aggregation time consumption for the entire network (cluster number = 5, fault tolerance = 1/4).

**Figure 14:**
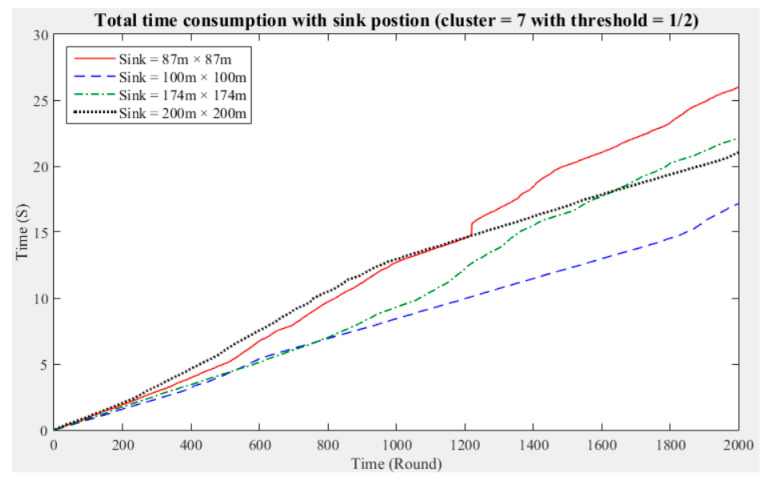
Data aggregation time consumption for the entire network (cluster number = 7, fault tolerance = 1/2).

**Figure 15:**
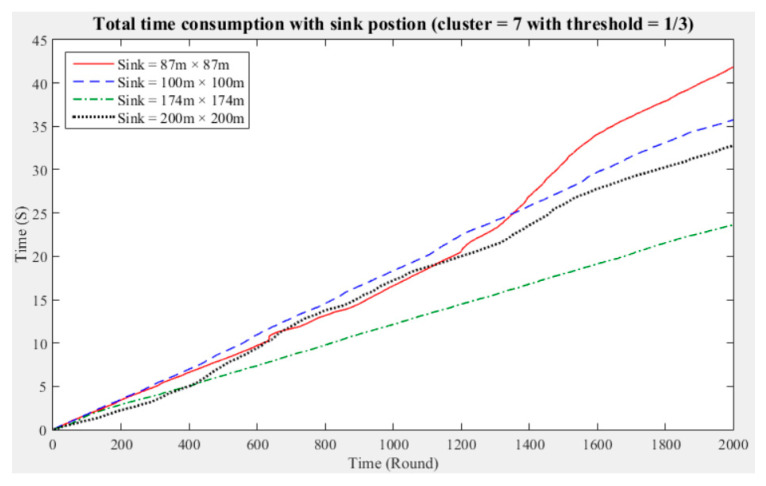
Data aggregation time consumption for the entire network (cluster number = 7, fault tolerance = 1/3).

**Figure 16:**
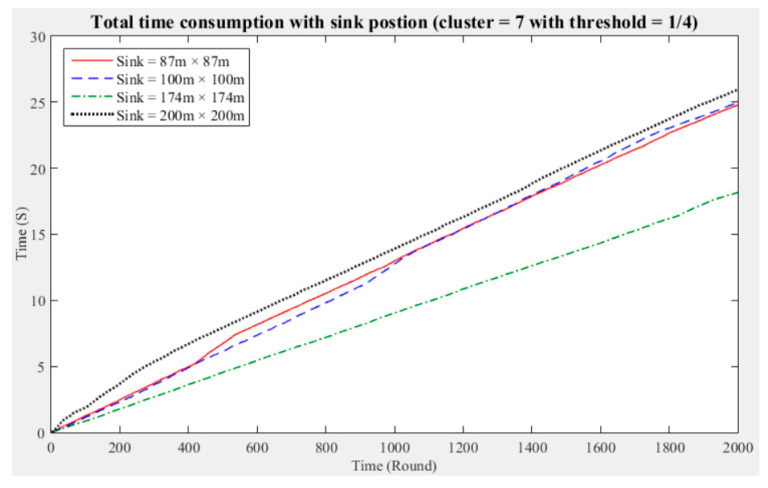
Data aggregation time consumption for the entire network (cluster number = 7, fault tolerance = 1/4).

To understand the manner in which different sink deployment positions may affect the time consumed by the entire network to aggregate data, we considered the above experimental results showing the three cluster performance worse than others. Then, we conducted experiments with five and seven clusters while considering different fault tolerance values. The results are presented in Figure 11, Figure 12, Figure 13, Figure 14, Figure 15 and Figure 16, and the time consumption is shown in Table 4.

Taking five clusters as an example, when the sink is located at 87 m × 87 m, the data aggregation time of the entire network is longest; when the fault tolerance values are one-half and one-quarter and the sink is located at 174 m × 174 m, the data aggregation time is shortest; and when the fault tolerance value is one-third and the sink is located at 200 m × 200 m, the data aggregation time of the entire network is shortest. With five clusters, sensing data aggregation requires the least time when the sink is located at 174 m × 174 m.

Taking seven clusters as an example, when the fault tolerance values are one-half and one-third and the sink is located at 87 m × 87 m and when the fault tolerance value is one-quarter and the sink is located at 200 m × 200 m, the data aggregation time of the entire network is longest. In contrast, when the fault tolerance values are one-third and one-quarter and the sink is located at 174 m × 174 m and when the fault tolerance value is one-half and the sink is located at 100 m × 100 m, the data aggregation time of the entire network is shortest. With seven clusters, sensing data aggregation takes the least time when the sink is located at 174 m × 174 m.

The above experimental results show that different sink deployment positions can affect the data aggregation time consumed by the entire network. For a clustered network, the positions of the sensor nodes, CHs, and sink, as well as the distances among them, will all result in a different data aggregation and transmission time. Table 4 shows that the deployment strategy of the sink, CHs, and sensor nodes established in accordance with the entire network environment will generate the most favorable outcomes in terms of the node energy consumption and data transmission time.

#### 4.2.4. Comparison with Other Research Methods

**Figure 17:**
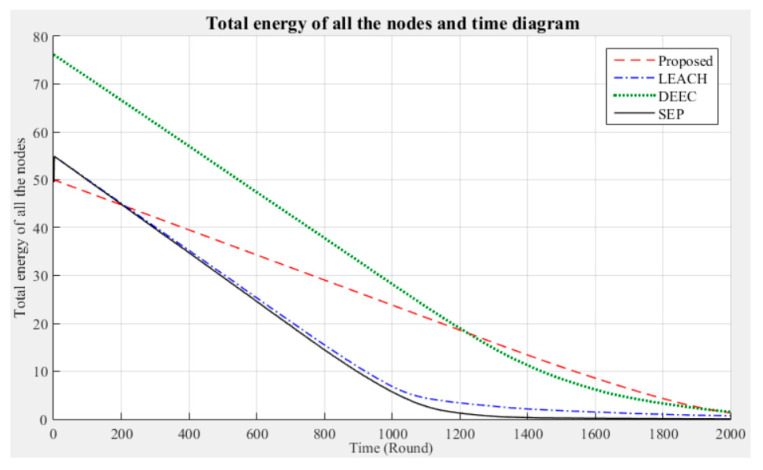
Entire network life cycle (no fault tolerance).

**Figure 18:**
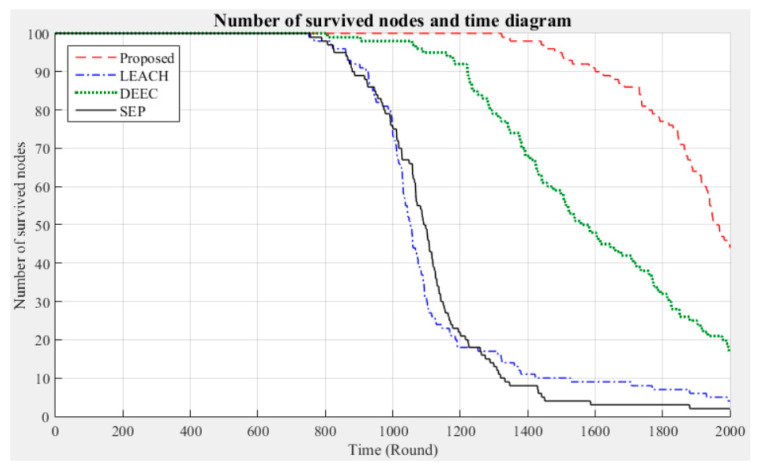
Number of surviving nodes in the network (no fault tolerance).

In addition, 2000 rounds of experiments were performed on 100 nodes using the proposed method and the LEACH, DEEC, and SEP algorithms; note that in these experiments, seven clusters were employed and fault tolerance was not considered. The corresponding changes in the network life cycle and number of surviving nodes were analyzed and compared; the results are shown in Figure 17 and Figure 18, respectively.

The experimental network life cycle results are shown in Figure 17. First, the algorithms were given different total energies at the beginning, because of the differences in their energy settings. The reasons are as follows:

- LEACH and SEP: Some sensor nodes are randomly elected to become advanced nodes and have more energy (1 J), and the other nodes become normal nodes and have initial energies of 0.5 J.
- DEEC: All the nodes, which are randomly set up, will have energies between 0.5 J and 1 J, according to their energy weight values.
- Proposed method: All the nodes have the same initial energy (0.5 J).

When the sensor network is operated using the LEACH, DEEC, or SEP algorithm, the total energy consumption of the nodes rapidly increases with the number of rounds. In contrast, when the proposed method is used, the total energy consumption tends to be stable. Moreover, from the differences in the number of surviving nodes after 2000 rounds of experiments, as shown in Figure 18, it can be seen that the proposed method can maintain a better node survival rate in the simulated experimental environment.

The results obtained in this portion of the study prove that the proposed method has much better performance in terms of node energy consumption and survival time, which can effectively extend the operating performance of the entire network. LEACH, DEEC, and SEP focus on cluster division and CH selection to reduce the energy consumption of the sensor nodes. This approach is likely to increase the time required for the nodes to join a cluster and select the CH. In contrast to these methods, the proposed approach adopts a different process. Firstly, the original signals acquired by the sensor nodes in each cluster are used to define a threshold and are asserted. Then, the cluster majority results are calculated by a fixed number of CHs. Finally, the network consensus is calculated by the sink node. According to the Byzantine consensus-based sensing results, the CHs aggregate the sensor data of each cluster and transmit them to the sink. This approach avoids the large energy consumption of sensor nodes due to frequent data transmission during network operation. Moreover, the CHs only send binary data during the calculation process, avoiding excessive energy consumption. Compared with LEACH, DEEC, and SEP, in which the CHs are frequently replaced and the network load is increased, if fixed and high-power CHs can be appropriately deployed in each cluster, the operating efficiency of clustered networks can be maximized.

## 5. Conclusions and Future Research

The objective of this study was to improve the data aggregation efficiency of WSNs via node cooperation. Based on the Byzantine fault tolerance mechanism, the principle of majority decision, and the characteristics of the MC algorithm, the sensor data are continuously acquired in each cluster; then, consensus is rapidly determined and data aggregation is completed when the number of faulty nodes is lower than the allowed fault tolerance. Furthermore, the experimental results show that a multi-cluster network architecture with an appropriate fault tolerance value can improve the success rate of reaching a data consensus and sending data to the sink. However, using a clustered network architecture for sensor data aggregation and having too many clusters and CHs will increase the difficulty of distributed network management and consensus calculation, which may lead to a non-deterministic polynomial problem.

Based on the results of the series of experimental analyses presented in this paper, this current study has produced three main findings. First, the introduction of the Byzantine fault tolerance mechanism can prevent faulty nodes from affecting the normal network operation and improve the network life cycle. Second, using the MC algorithm— which makes assertions with binary values of one and zero—can decrease the energy consumption of sensor nodes and reduce the data aggregation time. Third, reference indicators are provided to researchers such that they can assess data reliability and validity in distributed network environments.

There are two directions for future research. First, experimental data analysis and comparisons can be performed based on the proposed method and other common clustering algorithms employed in WSNs to verify the CH selection strategy and energy consumption fluctuations in a clustered network environment. Second, different fault tolerance mechanisms can be adopted to aggregate sensor data in the network to analyze the advantages and disadvantages of the BCA proposed in this paper.

## Figures and Tables

**Table 1:** Energy-efficient distributed protocols in wireless sensor networks (WSNs).

Method	Network Type	Characteristic
LEACH [8]	Clustering-based	CH election based on the residual energy of each node
DEEC [9]	Clustering-based	CH election based on the ratio between the residual energy of each node and the average energy of the network
SEP [10]	Clustering-based	CH election based on the weighted election probabilities of each node
PEGASIS [11]	Chain-based	Based on forming chains of sensor nodes and using multi-hop transmission
HEED [12]	Clustering-based	CH election based on residual energy of each node and each iteration

**Table 2:** Fault tolerance mechanisms compiled in this study.

Author	Fault tolerance model
Lamport et al. [19]	*f ≤* ⌊*n −* 1/3⌋
Moniz et al. [20]	*f ≤ n/*3 and *σ* ≤ ⌈*n* *− t/*2⌉*(n* *− k* *− t) + k* *−* 2
Rabin, MO [21]	*t ≤ n/*4

Note: *f* is the number of faulty processes, *n* is the total number of processes, *σ* is the number of omission faults, *t* is the number of actually faulty processes, and *k* is the number of processes required to reach a consensus.

**Table 3:** Experimental simulation parameters.

Simulation Parameters	
Transmit/receive electronics	50 nJ/bit
Energy for data aggregation	5 nJ/bit/signal
Number of sensor node	100
Sensor field (m^2^)	200 × 200
Sink location (*x*, *y*)	(87, 87), (100, 100), (174,174), (200,200)
Sensor node initial energy	0.5 J
CH initial energy	5 J
Radio region (M2)	87 × 87
Cluster number	3, 5, 7
Fault tolerance	1/2, 1/3, 1/4

**Table 4:** Time consumption with different sink positions.

Cluster	Fault Tolerance	Sink Position			
		87 m × 87 m	100 m × 100 m	174 m × 174 m	200 m × 200 m
5	1/2			✓	
	1/3				✓
	1/4			✓	
7	1/2		✓		
	1/3			✓	
	1/4			✓	

Note: the symbol (✓) represents the lowest time consumption when aggregating sensed data.
